# Where Will the Empire State Stand?

**Published:** 1912-08

**Authors:** 


### Where Will the Empire State Stand?

LAST year, in the *Medical Times* of New York, we commended the action of the University of Buffalo, in taking the initial step toward securing a higher matriculation standard. Allusion was made to the same matter in the September, 1911, issue of this JOURNAL. The following, from the *Bulletin* of the Illinois State Board of Health, needs no comment.

"Colorado now requires that a student who was matriculated in 1908-09, and graduated in 1912, shall have had one year of college work as preliminary training. This requirement is increased to two years in the case of students matriculated in 1910-11, and graduated in 1914.

The Colorado requirement, however, will not be imposed on those students, or practitioners, who take the examination of the Colorado State Board of Medical Examiners. It applies only to those receiving their licenses on credentials from other States.

The other States exacting one or more years of college work are as follows:

| State | Years | In force | Affecting graduates of |
|---|---|---|---|
| Connecticut ..................... | 1 | 1910 | 1914 |
| Indiana ......................... | 1 | 1910 | 1913 |
| Indiana ......................... | 2 | 1911 | 1914 |
| Iowa ............................ | 2 | 1911 | 1915 |
| Kansas .......................... | 1 | 1910 | 1914 |
| Minnesota ....................... | 2 | 1908 | 1912 |
| North Dakota .................... | 2 | 1908 | 1912 |
| South Dakota .................... | 2 | 1907 | 1911 |
| Utah ............................ | 1 | 1913 | 1917 |

In Utah the advance requirements are specifically provided for by law. In the other States mentioned they have been prescribed by the rules of the State medical board.

